# Case report: One case of acute myeloid leukemia M3 with atypical morphology

**DOI:** 10.3389/fonc.2024.1341840

**Published:** 2024-03-19

**Authors:** Fa-Sheng Liu, Hua-Liang Huang

**Affiliations:** Department of Laboratory, Inner Mongolia Baogang Hospital, Baotou, China

**Keywords:** acute promyelocytic leukemia, atypical, diagnosis, flow cytometry, chromosome analysis

## Abstract

Acute promyelocytic leukemia (APL) is a type of acute myeloid leukemia. About 2% of APL is characterized by atypical rearrangements. Here we reported one APL case with atypical manifestations and morphology. A 35-year-old woman patient, mainly due to fatigue, poor appetite for over 10 days and intermittent fever for 3 days. combined with the results of flow cytometry, fusion gene and chromosome, the patient was diagnosed as AML-M3 with atypical morphology. Double induction therapy with retinoic acid and arsenous acid was immediately administrated. Idarubicin was administrated on the 18th day. A re-examination was performed in the 5th week, both the blood routine test and myelogram showed normal results, and the fusion gene turned negative, indicating complete remission. When atypical morphology occurs, peripheral blood POX staining may be performed to check the abnormal cells. Flow cytometry, chromosome analysis, and fusion gene analysis are also required for further diagnosis.

## Introduction

Acute promyelocytic leukemia (APL) is a type of acute myeloid leukemia (AML) characterized by malignant proliferation of abnormal promyelocytes and the PML-RARA fusion gene, accounting for 10-15% of newly diagnosed AML (1). APL is often common in males in their prime. APL has aggressive manifestations such as severe bleeding and possible complication of disseminated intravascular coagulation (DIC), with intracranial hemorrhage being the primary cause of death.

APL has the genotype of t (15; 17) (q24; q21) balanced translocation. AML patients with a complex karyotype (CK) account for 10 ~ 12% of all AML patients and are associated with a poor prognosis (2). CK is defined as the karyotype having ≥3 chromosomal abnormalities, with the most common unbalanced abnormality causing loss of material in the chromosome arms of 5q, 7q and/or 17p. Abnormalities in 5q, 7q and/or 17p indicate the typical CK, otherwise the atypical CK (3). About 2% of APL is characterized by atypical rearrangements, the t (15; 17) (q24; q21) chromosomal translocation and PML-RARα fusion gene are cytogenetic and molecular genetic features of typical APL patients. However, some APL patients lack typical translocations, and no typical t (15; 17) has been identified by conventional karyotyping, but PML-RARα gene rearrangements can be detected at the molecular level, usually due to variant translocations of APL. Variant translocations of APL can be divided into three types: simple variants: translocations between chromosomes 15 or 17 and another chromosome; complex variants: involving at least three or > three chromosomes including chromosomes 15 and 17; occult variants: patients with typical clinical manifestations, cytomorphological features, and immunophenotypes of APL, did not reveal typical t (15; 17) (q24; q21) translocations using conventional cytogenetic techniques, but the presence of submicroscopic PML and/or insertions or more complex rearrangements of the RARα gene resulting in PML-RARα transcription was found after analysis by molecular techniques (4, 5). The molecular characterization of atypical APL is not currently clear which easily lead to misdiagnosis. Genetic features confirmation for APL patients is required and should be performed within a 24-to 48-hour referral period to ensure rapid initiation of treatment and thereby reduce bleeding complications and early mortality (6). Here we experienced one APL case with atypical manifestations and morphology, as below.

## Case description

The patient, a 35-year-old woman, was admitted to the hospital on March 7, 2022, mainly due to fatigue, poor appetite for over 10 days and intermittent fever for 3 days. She complained of fatigue and poor appetite after catching cold 10 days ago, accompanied by dizziness, headache, nausea and vomiting several times. She took cold medicine but did not improve. 3 days before admission, she developed intermittent fever with the highest body temperature of 38°C. Then she went to the community health clinic for intravenous antibiotics (Azithromycin, 0.5g/day) for 2 days but did not improve. Blood routine tests showed a significant increase in white blood cells, accompanied by anemia and reduced blood platelet, and archeocytes and juvenile cells were observed in peripheral blood. For further diagnosis and treatment, she visited the Hematology Department. She had no history of blood disease and no family history related. She underwent surgery on the left breast due to breast cancer 5 years ago, followed by postoperative radiotherapy and chemotherapy. Currently, she takes exemestane 25mg/d orally and goserelin subcutaneously every 3 months.

Physical examination when admission: anemic appearance; no bleeding spots, ecchymosis or rash in the skin and mucosa; no swelling of superficial lymph nodes; positive sternum tenderness; pharyngeal congestion; thick breathing of both lungs and no rales; untouched liver and spleen; and no edema of both lower limbs.

Lab tests:

Blood routine test: WBC:160.04×10^9^/L↑, HB:100.0g/L↓, PLT:65.0×10^9^/L↓. Peripheral blood cell morphology indicated a significantly decreased rate of neutrophils to lymphocytes, with abnormal cells accounting for 97% and abnormal promyelocytes being considered.

Coagulation function: PT: 15.5s ↑; APTT: 24.2s; TT: 20.1 s; FIB: 0.900g/L↓; D-dimer: 8.86μg/Ml; FDP: 43.48μg/mL↑.

**Figure 1:**
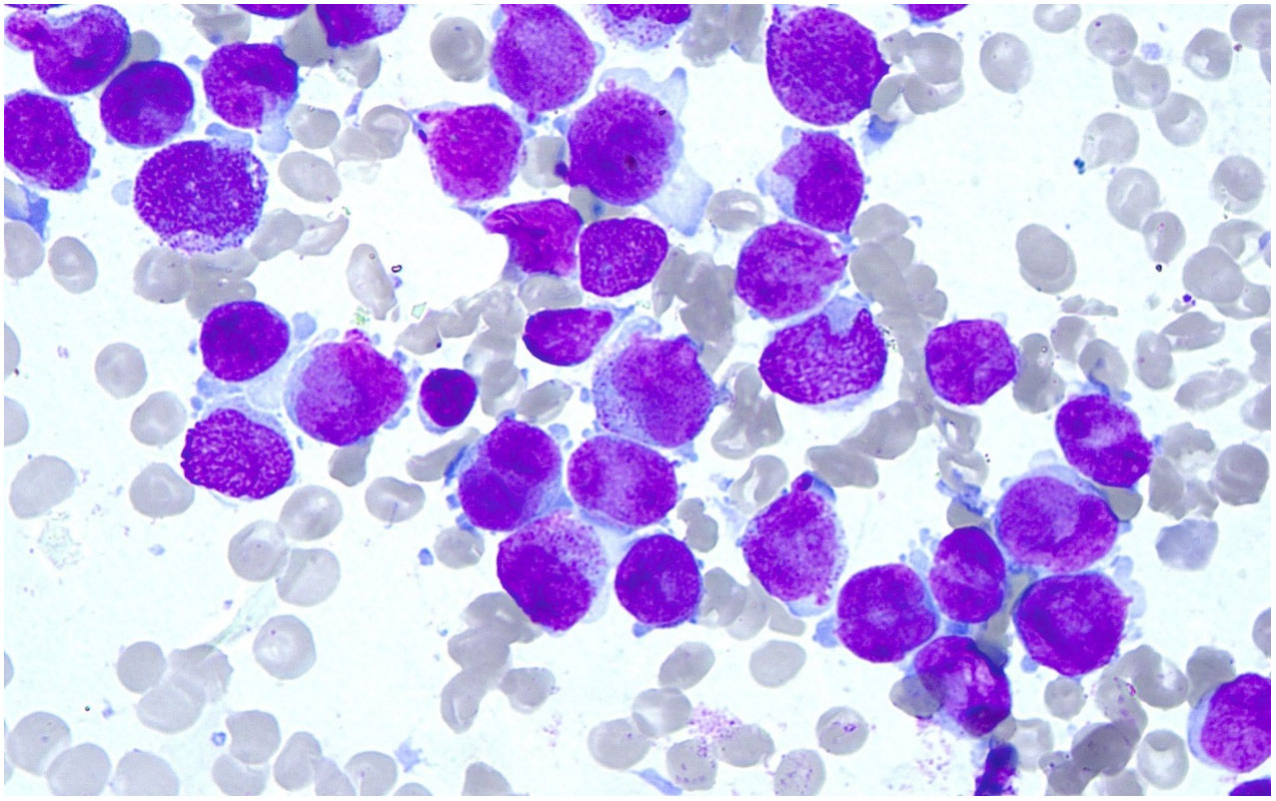
Results of Wright’s stain. Wright’s stain showed the marrow morphology.

Marrow morphology: the cells showed obviously active proliferation, with the G: E (granulocytes to erythrocytes) ratio of 118:1; granulocytes accounted for 94.4%, showing a significant increase. Atypical abnormal promyelocytes were found to account for 92.8%, which had relatively regular bodies and nucleus, and some natiform or butterfly-shaped nucleus were occasionally observed. The cells had less cytoplasm, few or no granules, less inner and outer cytoplasm in individual cells, and occasional Auer bodies. Erythroid cells accounted for 0.8%, and marrow proliferation was significantly inhibited. Mature erythrocytes had a roughly normal morphology. Lymphocytes accounted for 4.8% and had a roughly normal morphology. 1 megakaryocyte was observed, and platelets were visible. No other special cells were observed (**Figure 1**).

**Figure 2:**
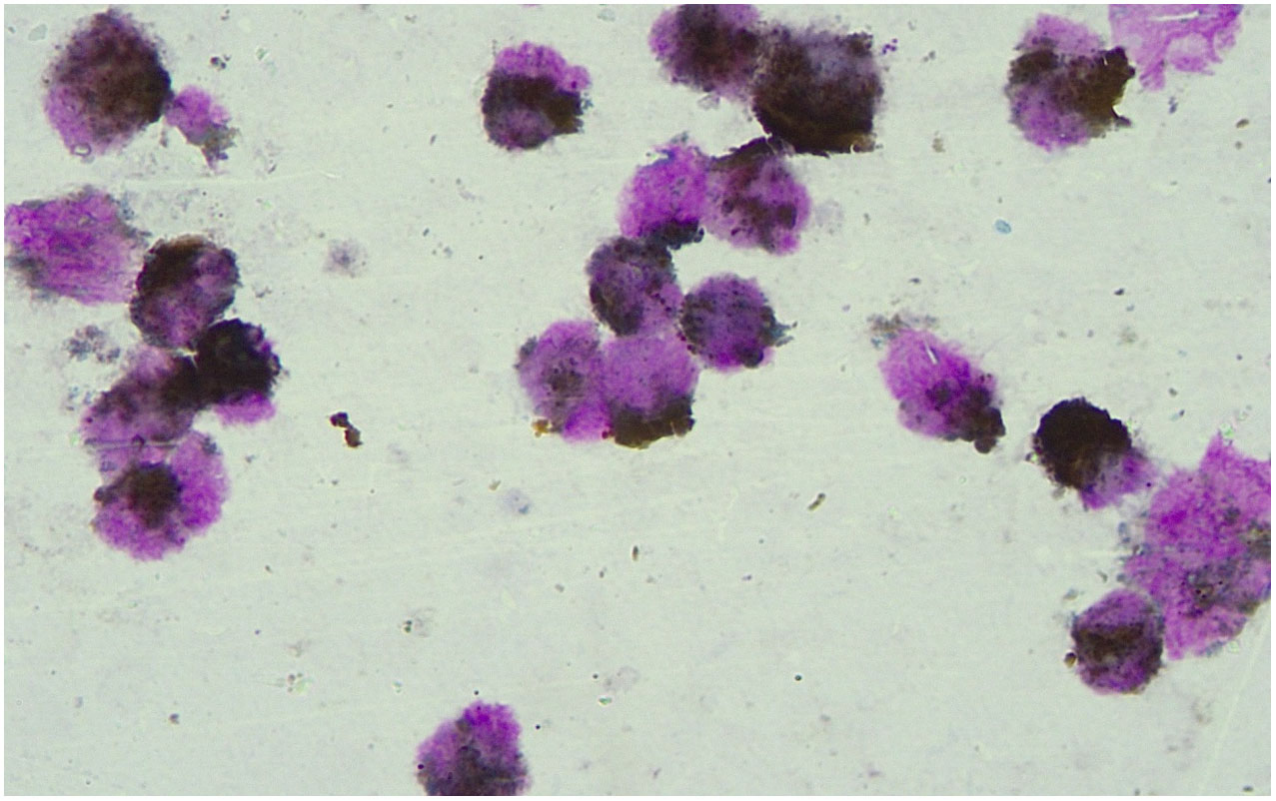
Results of peroxidase POX staining. Peroxidase POX staining showed the marrow morphology which indicated the AML-M3 with atypical morphology.

Homeochemical staining of peroxidase POX (+ ~ + +) (**Figure 2**). Based on the morphological findings and cell histochemical staining results above, the patient was considered to have AML-M3 with atypical morphology.

In view of the high WBC and atypical cell morphology findings, clinical detection was rapidly made. Since the patient’s manifestations, coagulation, and marrow morphology were significantly different from those suffering from the typical APL M3, further fusion gene, flow cytometry test, and chromosome examination were required to confirm the diagnosis. Thus, hydroxyurea (1.0, tid, oral) was initially administrated to reduce white blood cells, meanwhile, hydration, urine alkalinization, anti-infection, and supportive treatment were also performed. Routine blood tests and coagulation were closely monitored, and flow cytometry, fusion gene and chromosome examination results were awaited synchronously.

**Figure 3:**
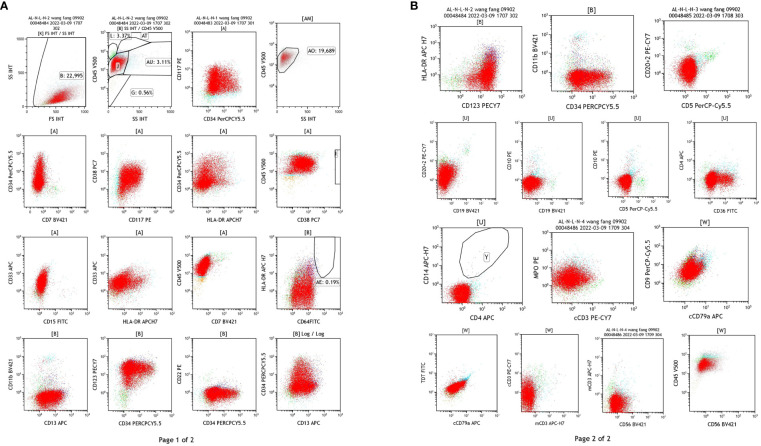
Results of flow cytometry test of the blood samples. **(A)** The detection of CD34, CD177, HLA-DR, CD33, CD13, CD11b, CD64. **(B)** The detection of CD20 + 2, CD15, CD19, CD5, CD79a, CD56.

Flow cytometry: Abnormal myeloid cells were observed, accounting for 85.62% of the nuclear cells, expressing CD9 and MPO, heterogeneous expressing CD13, and not expressing HLA-DR. AML was considered, and AML with PML/RARa could not be excluded (**Figure 3**). (In this case, SSC was small, non-specific fluorescence was not obvious, and some cells were CD34 positive, so APL of fine or less granule should be excluded.)

Fusion gene: PML/RARa S had the copy number 171048, and the quantitative result of the fusion gene was 37.36%.

**Figure 4:**
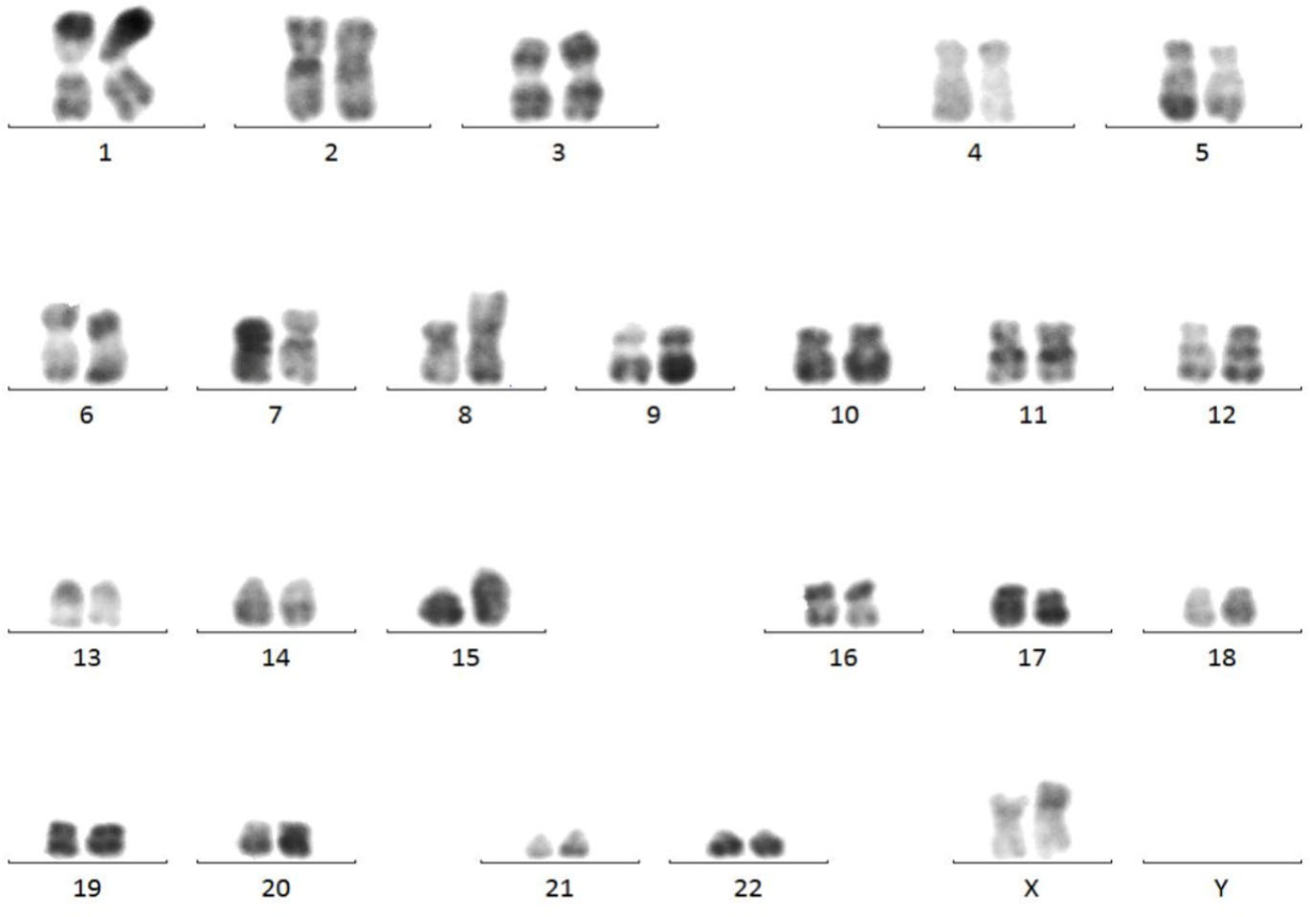
Results of marrow karyotype analysis. Karyotype analysis showed the clonal abnormalities add (8p), t (15; 17).

Marrow karyotype analysis: The karyotype was 46, XX, add (8) (p12), t (15; 17) (q24; q21) (6), having clonal abnormalities add (8p), t (15; 17) (**Figure 4**).

In summary, combined with the results from flow cytometry, fusion gene and chromosome, the patient was diagnosed as AML-M3 with atypical morphology. Double induction therapy with retinoic acid (20mg, orally, bid) and arsenous acid (10mg per day, iv drip for 32 days) was immediately administrated. Idarubicin was administrated on the 18^th^ day. A re-examination was performed in the 5^th^ week, both the blood routine test and myelogram showed normal results, and the fusion gene turned negative, indicating complete remission.

## Discussion

Typical APL often manifests severe bleeding, decreased red blood cells, white blood cells and platelets in peripheral blood, abnormal coagulation function, and easy induction of DIC in the early stage, and secondary fibrinolytic hemorrhage. Abnormal promyelocytic proliferation with increased granules was predominant in the bone marrow (>30% of non-erythroid nucleated cells). Flow cytometry showed CD34 (-) and DR (-), and most of the APL fusion genes were PML/RARa L-type (7–9).

Literature has shown that AML-M3 with atypical morphology accounts for only 0.80% ~ 1.08% of APL (10). Different from typical M3 patients, their peripheral red blood cells and platelets were mostly decreased, while the white blood cells were significantly increased, which can reach more than 100×10^9^/L. Their coagulation function was slightly abnormal. The marrow morphology showed abnormal promyelocytes, relatively neat cell bodies, fewer cytoplasm granules, occasional butterfly-shaped and natiform nuclei, visible Auer bodies, and no obvious inner and outer cytoplasm. The APL fusion gene was PML/RARa type S (11–13). Clinical manifestations could rarely induce DIC and less bleeding. Atypical APL usually has an immunophenotype like the typical APL, but there are significant differences in manifestations and morphology, including changes in whole blood cells, symptoms related (weakness, fatigue, infection), and bleeding (14). Currently, all-trans retinoic acid (ATRA) and arsenic trioxide (ATO) are primarily administrated for APL in clinical practices. However, the atypical CK-APL disease subtype was less likely to respond to more effective regimens in APL patients with typical TP53 mutations, such as those with inv (3) (q21q26) or t (3; 3) (q21; q26) 6-9 is one such subgroup that has a poor response to ATRA therapy (12). Clinically, it is crucial to develop new therapies for APL (especially chemotherapy-resistant subtypes) disease subtypes. Meanwhile, different disease subtypes and corresponding treatments should be considered when searching for potential therapeutic targets.

In this study, atypical abnormal promyelocytes were found in the bone marrow of 92.8% of the patients, and their cell bodies and nuclei were relatively regular, with occasional partial maternal or butterfly nuclei. Cells had less cytoplasm, few or no granules, less cytoplasm inside and outside individual cells, and occasional Auer bodies. The patient was considered AML-M3 with atypical morphology based on assimilation staining of peroxidase POX. Because the cell morphology was not typical, fusion gene, flow cytometry, chromosome examination were used to confirm the diagnosis.

After the patient was diagnosed with morphologically atypical AML-M3, the patient’s symptoms were totally relieved after double induction therapy with retinoic acid and arsenous acid, showing no obvious bleeding and basically normal coagulation. Atypical abnormal promyelocytes in bone marrow morphology are likely to be recognized as myeloid blasts, and this disease is easily misdiagnosed as other acute myeloid leukemias. Thus, persons engaged in cell morphology analysis are required to have solid knowledge of cell recognition and rich experience in smears interpretation to better serve the clinical practice. When diagnosing marrow cell morphology, the cells should be identified carefully, and the changes in the nucleus and cytoplasm should be observed to find the differences. Meanwhile, gradually accumulating experience can ensure the accuracy of diagnosis.

## Conclusions

When atypical morphology occurs, peripheral blood POX staining may be performed to check the abnormal cells. Flow cytometry, chromosome analysis, and fusion gene analysis are also required for further diagnosis.

## Data availability statement

The raw data supporting the conclusions of this article will be made available by the authors, without undue reservation.

## Ethics statement

The studies involving humans were approved by the ethics committee of Inner Mongolia Baogang Hospital. The studies were conducted in accordance with the local legislation and institutional requirements. The participants provided their written informed consent to participate in this study. Written informed consent was obtained from the individual(s) for the publication of any potentially identifiable images or data included in this article.

## Author contributions

F-SL: Conceptualization, Formal analysis, Project administration, Visualization, Writing – original draft, Writing – review & editing. H-LH: Conceptualization, Formal analysis, Project administration, Visualization, Writing – original draft, Writing – review & editing.
